# Rapid On-Site Formation of a Free-Standing Flexible Optical Link for Sensing Applications

**DOI:** 10.3390/s16101643

**Published:** 2016-10-05

**Authors:** Carlos Angulo Barrios

**Affiliations:** 1 Instituto de Sistemas Optoelectrónicos y Microtecnología (ISOM), ETSI Telecomunicación, Universidad Politécnica de Madrid, Ciudad Universitaria s/n, 28040 Madrid, Spain; carlos.angulo.barrios@upm.es; Tel.: +34-91-549-57-00; 2 Department of Photonics and Bioengineering, ETSI Telecomunicación, Universidad Politécnica de Madrid, Ciudad Universitaria s/n, 28040 Madrid, Spain

**Keywords:** optical sensors, micro-optical devices, plastic waveguides, diffraction gratings, guided waves, liquid detection, optical links

## Abstract

An optical link, based on a conventional Scotch tape waveguide, for sensing applications requiring rapid on-site assembly is proposed and demonstrated. The flexible waveguide contains an integrated aluminum one-dimensional grating coupler that, when stuck on the radiative surface of a light emitting device, allows light to be coupled in and transmitted through the tape, whose tip end is, in turn, adhered onto the photosensitive surface of a photodetector. The (de)coupling approaches exhibit high alignment tolerances that permit the formation of a free-standing flexible optical connection between surface-normal optoelectronic devices without the need of specialized equipment. As the first demonstration of a sensing application, the proposed optical link is easily configured as a cost-effective intensity-based refractometric sensor for liquid detection, which can be applicable to on-site quality and process control of, for example, beverages.

## 1. Introduction

Free-standing optical waveguides offer appealing characteristics that make them well suited to numerous sensing applications. For example, the waveguide surface exposed to the environment is increased as compared to a waveguide fabricated on a support, which is favorable for enhancing the device sensitivity to parameters of the surrounding medium through evanescent field interaction [1,2]. Besides, free-standing waveguides are relatively free to move around its equilibrium position, which makes them highly adequate for implementing movement optical sensors [3,4]. If high flexibility, low-cost and robustness are added to these features, the versatility and range of sensing applications of this type of waveguiding devices increase significantly. Plastic optical fibers (POFs) are probably the most representative example of the successful application of flexible free-standing waveguides to the sensors field [5,6].

An additional feature that could further extend the applicability of these optical sensors is the feasibility for rapid in-situ formation of optical connections in a cost-effective and easy manner, that is, without requiring specialized instrumentation and/or qualified personnel. This should allow the implementation of ready solutions for a variety of field applications based on compact sensing systems. In the case of POFs, fiber connection to light emitters and receivers is achieved via the butt coupling method. Although the large core diameters of POFs (typically, 0.5 mm–1 mm) facilitate light coupling, specialized fiber termination procedures (such as cutting and polishing) and tools are required, and bulky connectors or focusing discrete optical elements are used as link terminations [6]. This makes the establishment of rapid connections by untrained (non-technical) users or without the proper apparatus difficult.

A key issue to facilitate the formation of fast on-site optical links based on waveguides is alignment-tolerant surface mount assembly between the waveguide tip ends and optoelectronic devices (light emitting diodes (LEDs), laser diodes and photodetectors). Light coupling via a diffraction grating [7] is particularly suitable for obtaining out-of-plane high alignment tolerances and the planar geometry of the gratings makes them compatible with wafer or board scale batch planar manufacturing processes. This geometrical characteristic makes its use especially appropriate for coupling light into planar waveguides. In a recent work [8], the first results on light coupling into a conventional pressure-sensitive adhesive (PSA) Scotch tape waveguide, by means of a nanohole array metal grating coupler (GC) integrated in the flexible plastic tape, were presented, introducing it as a promising solution for implementing cost-effective and versatile adaptive optical links.

**Figure 1:**
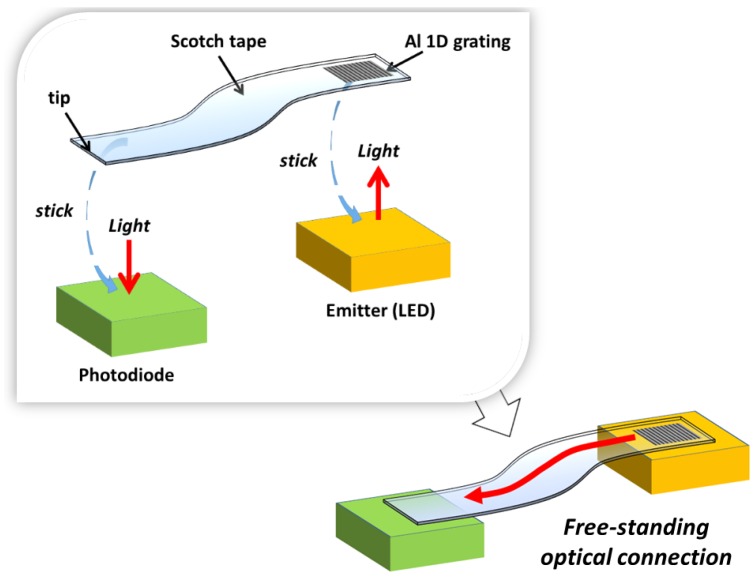
Schematics of the proposed rapid on-site establishment of an optical link between a vertical emitting device (an LED) and a vertical detecting device (a photodiode) through a flexible tape waveguide with an integrated aluminum one-dimensional (1D) grating coupler.

In this work, the feasibility to form a rapid on-site optical configuration based on both, a free-standing Scotch tape waveguide with integrated GC and low-cost optoelectronic devices is evaluated, and the first sensing application of the resulting cost-effective easy-to-implement optical system is reported. The proposed scheme is illustrated in Figure 1. A surface-normal emitter (e.g., a vertically emitting LED) and a surface-normal photodetector (e.g., a photodiode (PD)) are optically connected through a PSA tape waveguide containing a one-dimensional (1D) aluminum GC in one of the waveguide terminals, which is stuck on the emitter radiative surface. The other waveguide terminal (tip) is attached to the photosensitive surface of the PD. The GC allows light emitted vertically to be coupled into the tape waveguide. A 1D GC is used in this work, instead of the two-dimensional (2D) geometry reported in [8], in order to improve coupling into the waveguide direction of interest [9]. Guided light exiting the waveguide tip interacts with the PD, which converts the optical power into an electrical current. The following key characteristics are analyzed in the paper: alignment tolerance between the waveguide coupling elements and the corresponding optoelectronic devices, optical loss due to tape bending and dynamic response of the studied optical system. Finally, an intrinsic intensity-based sensor for fluid detection relying on tape waveguide macrobending is easily configured and demonstrated as an example of a sensing application.

## 2. Materials and Methods

**Figure 2:**
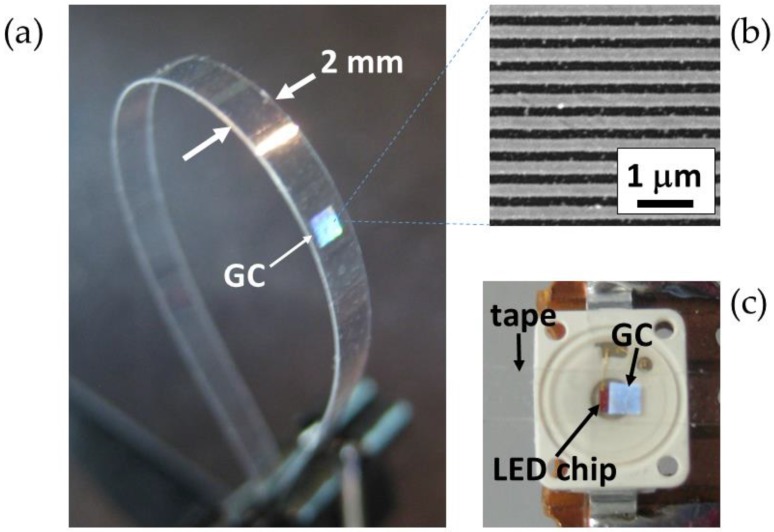
(**a**) Photograph of a fabricated tape waveguide with an integrated 1.2 mm × 1.2 mm 1D Al grating coupler (GC); Al stripes are normal to the tape length direction. (**b**) Scanning electron microscope (SEM) image of the Al GC (period = 500 nm, Al stripe width = 270 nm). (**c**) Top view photograph of a GC integrated in a tape waveguide placed on the emitting surface of a packaged LED to couple light from the LED chip into the tape waveguide. The LED chip dimensions are approximately 1 mm × 1 mm.

An optical waveguide made of a conventional 50-µm-thick Scotch tape (#550 Scotch®, 3M, St. Paul, MN, USA) with an integrated Al 1D GC was fabricated as described in [9]. In short, a 1.2 mm × 1.2 mm Al wire grid (period = 500 nm, Al stripe width = 270 nm, Al thickness = 100 nm) was first fabricated on a polycarbonate substrate [10] and transferred to a Scotch tape by a simple *stick-and-peel* procedure [11], the stripes being perpendicular to the tape length direction. Then, another piece of the same type of Scotch tape was stuck on the former, embedding the GC between two tapes. Finally, the double tape was cut longitudinally in order to form a 2-mm-wide 100-µm-thick flexible waveguide with an integrated GC as shown in Figure 2a.

A red (λ_peak_ = 632 nm) vertically emitting InGaAlP-based LED (OSRAM Opto Semiconductors, LR W5SM-HZJZ-1-1, Regensburg, Germany) and a silicon pin photodiode (OSRAM Opto Semiconductors, BPW 34, Regensburg, Germany) were used as the optoelectronic emitter and receiver, respectively. The operation wavelength of 632 nm is well within the spectral coupling bandwidth (500 nm–725 nm) of the embedded grating [9]. The LED chip has dimensions of approximately 1 mm × 1 mm, exhibits Lambertian radiation characteristics and a spectral bandwidth (full width at half maximum) of 18 nm, and is encapsulated in a 6 mm × 7 mm × 2 mm surface-mount device (SMD) package. The photodiode responsivity at λ = 632 nm is 0.434 A/W, the dimensions of its radiant sensitive area are 2.65 mm × 2.65 mm and has a directivity of ±60° (half-angle). These particular optoelectronic components were chosen because of their dimension compatibility (the LED chip has similar dimensions and shape as the GC, and the PD is slightly larger than the waveguide tip); the planar geometry of their encapsulations, which facilitates tape waveguide attachment; high performance; and low cost.

**Figure 3:**
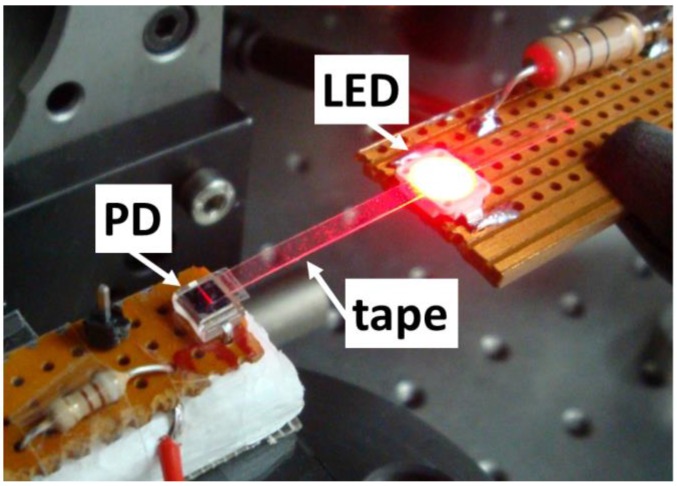
Photograph of the configuration and operation of the proposed flexible, adhesive tape-based optical link between a vertically emitting LED and a surface-normal photodiode (PD) for rapid on-site sensing applications. The tape waveguide integrates a 1D Al grating coupler (GC) that is placed on the LED radiative surface. The GC allows red light emitted by the LED to be coupled into the tape waveguide, whose tip end is attached to the PD detecting surface. The guided light reaches the PD where it is converted into an electrical signal. Red light propagation and scattering at the tip end on the PD are clearly observed.

The LED and PD were soldered on different PCBs, together with appropriate series resistors (10 Ω for the LED and 910 Ω for the PD) to create simple biasing electrical circuits. The optoelectronic devices were optically connected via the tape waveguide by placing the GC on the LED (Figure 2c) and the waveguide tip end on the PD surface as shown in Figure 3. A direct current (DC) power supply was used to forward/reverse bias the LED/photodiode. A transistor-transistor logic (TTL) pulse generator was employed to excite the LED for characterizing the dynamic response of the system. The PD response (photocurrent) was monitored by measuring the voltage drop in the corresponding series resistor with a digital multimeter (DC operation) or with a digital oscilloscope (pulsed operation).

## 3. Results

**Figure 4:**
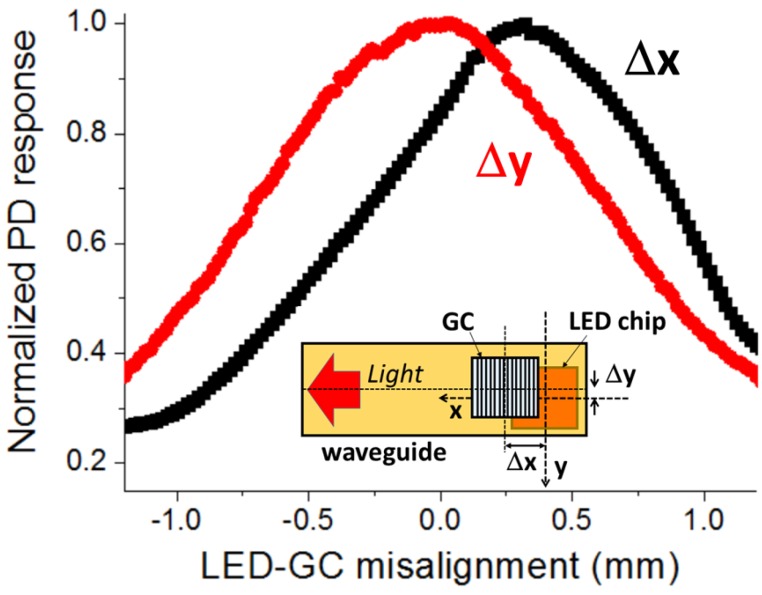
Normalized power detected by the photodetector (PD) as a function of the misalignment between the LED chip and the grating coupler (GC) along the x-axis and y-axis. Δx (Δy) equals the LED chip center position minus the GC center position along the x-axis (y-axis) according to the inset diagram. Guided light propagation direction is +x-axis.

Figure 4 plots the normalized PD response as a function of the LED-GC misalignment. Δx (Δy) is defined as the difference between the centers of the LED chip and the GC along the x-axis (y-axis) for Δy (Δx) = 0 (inset of Figure 4). Light propagates along the +x-axis. The misalignment curve is highly symmetric along the y-axis (normal to the waveguide length) with a maximum at Δy = 0. This is expected since the coupling variation along this axis should be related to the overlap between the LED chip area and that of the GC. A different behavior occurs along the x-axis (waveguide length direction), with the maximum being at Δx = 320 µm, not at Δx = 0. As studied in a recent work [9], the highest surface-normal coupling efficiency of the GC takes place when the incident beam is focused onto the grating edge closer to the desired guided light propagation direction. At Δx = 320 µm, the center of the LED chip, where the vertically emitted power is maximum, is placed under the grating side closer to the propagation direction (+x-axis), thus leading to the highest coupled power (PD response). That is, the GC should be positioned on the LED surface as shown in Figure 2c in order to maximize the coupled power in the tape waveguide towards the left direction. From Figure 4, the LED-GC alignment tolerance, defined as the misalignment range within which the coupled power between both elements is larger than 90% of the maximum coupling, is ±250 µm along both the x- and y-axis.

**Figure 5:**
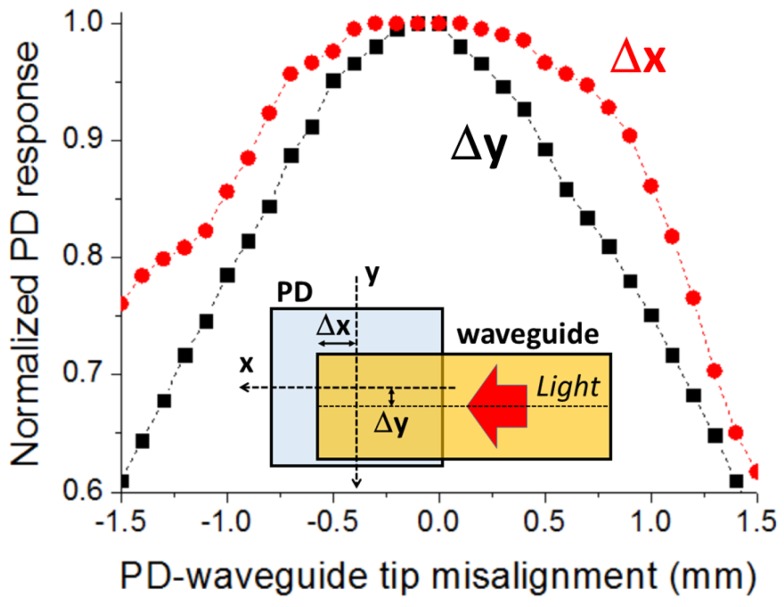
Normalized power detected by the photodetector (PD) as a function of the misalignment between the PD and the waveguide tip end along the x-axis and y-axis. Δx (Δy) equals the PD center position minus the tip center position along the x-axis (y-axis) according to the inset diagram. Guided light propagation direction is +x-axis.

Figure 5 illustrates the normalized PD response as a function of the PD-waveguide tip misalignment. Δx (Δy) is defined as the difference between the centers of the PD and the waveguide tip along the x-axis (y-axis) for Δy (Δx) = 0 (inset of Figure 5). Similarly to the LED-GC case, the misalignment curve along the y-axis is symmetric with a maximum at Δy = 0. Concerning the dependence along the x-axis, the highest value is also obtained for zero misalignment (Δx = 0). Additionally and remarkably, the Δx curve exhibits a relatively large flat region around the maximum, indicating a high alignment tolerance along the x-axis. In particular, the alignment tolerances, defined as those for the LED-GC case, along the x-axis and y-axis, are ±850 µm and ±500 µm, respectively. Both the LED-GC and PD-tip alignment tolerances are large enough to allow manual assembly to be carried out by a moderately skilled operator assisted by a simple magnifier. The attachment between the coupling elements and the corresponding optoelectronic devices can be achieved by using a variety of sticking methods such as a double-stick tape and/or an epoxy adhesive glue, the former being ideally suited for sensing applications requiring replaceable and disposable tape waveguides.

The amount of optical power detected by the photodiode (P_PD_) for a given optical power emitted by the LED (P_LED_), that is, the ratio (P_PD_/P_LED_), is:

(P_PD_/P_LED_) = η_tip_ η_GC_ exp(−α_w_ L_w_) (1)

where η_tip_ and η_GC_ are the coupling efficiencies between the waveguide tip and the PD and between the LED and the waveguide, respectively, and α_w_ and L_w_ are the waveguide loss coefficient and length, respectively. For the optimum LED-GC and PD-tip alignments, the measured ratio was (P_PD_/P_LED_) = 1.7 × 10^−3^, where P_PD_ was obtained by dividing the measured photocurrent by the PD responsivity. From a previous work [9], α_w_ = 0.072 mm^−1^ (3.1 dB/cm), whereas the length of the used waveguide was L_w_ = 16.35 mm. η_tip_ is the power sensed by the PD on which the tip is attached, divided by the total output power from the waveguide tip (recorded as indicated in [9]). For the optimum PD-tip alignment, measured η_tip_ = 0.30. Therefore, from Equation (1), η_GC_ = 0.018. The highest theoretical surface-normal coupling efficiency of the embedded GC that can be achieved is 25% under particular incidence beam conditions [9]: TM (E-field normal to the Al stripes)-polarized beam focused (spot size ~200 μm) close to the grating edge. Deviations from these conditions can decrease the coupling efficiency significantly due to rediffraction losses and GC polarization dependence. This explains the obtained low η_GC_ value since no focusing optics is used between the grating and the LED chip, which, in addition, emits unpolarized light. Still, the reduced coupling efficiency is made up for by the simplicity and high alignment tolerance of the LED-GC surface mount assembly.

A simple method to increase the ratio (P_PD_/P_LED_) is to place a piece of sheet of a reflecting material on both the GC over the LED, to enhance η_GC_, and the waveguide tip over the PD to raise η_tip_. In the LED-GC case, the reflecting sheet forces light transmitted through the GC to interact again with the grating, increasing the coupled power into the waveguide. In the PD-tip case, the reflecting material redirects the fraction of light that exits the waveguide and does not impinge directly on the PD towards the latter, increasing the detected power. For example, a ~(5 mm × 5 mm) piece of Al foil just placed on the GC-LED was measured to increase the coupled power by 82%, which is equivalent to boost the GC coupling efficiency to η_GC_ = 0.033. A similar Al foil piece placed on the tip-PD increased the detected power by 13%, that is, it raised the detection efficiency to η_tip_ = 0.34. Thus, by simply placing small pieces of Al foil on top of both optical link terminals, the measured ratio (P_PD_/P_LED_) = 3.5 × 10^−3^, which means an increase of 106% compared to the uncapped termination configuration.

**Figure 6:**
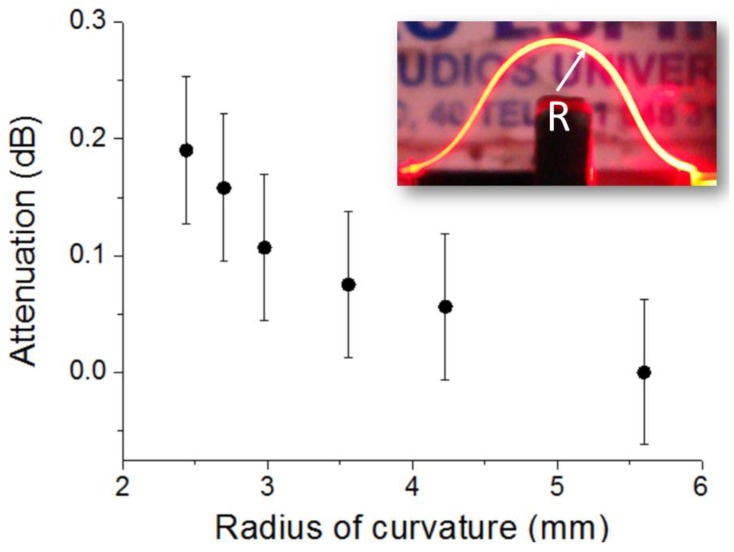
Measured optical attenuation introduced by bending a 24.7-mm-long tape waveguide at different radii of curvature (R). Inset shows a profile photograph of a bent tape waveguide guiding light, depicting the radius of curvature.

Adaptive tape bending is one of the most remarkable properties of flexible waveguides. To study this matter, bent waveguides were created using the configuration shown in Figure 3, by attaching the coupling elements (GC and tip) of a 24.7-mm-long waveguide to the corresponding optoelectronic devices and bringing them closer. An opaque vertical barrier was placed between the emitter and the receiver to avoid non-guided light detection. Figure 6 plots the measured loss as a function of the bent waveguide radius of curvature (R). Attenuation was calculated as 10 × log (P_R_/P_∞_), where P_R_ and P_∞_ are the detected powers for a radius of curvature R and for a straight tape (R = ∞), respectively. For R = 2.4 mm, the total bending angle between the LED and the PD is approximately 360°, which signifies a bending loss of (5.3 ± 1.7) × 10^−4^ dB/°. This low value is attributed to the large cross section of the fabricated tape waveguide, which makes it highly multimode at the operation wavelength. For the sake of comparison, bending loss (dB/°) of a 1 mm step-index POF for R = 3 mm has been measured to be one order of magnitude larger [12] than that of the studied tape waveguide. Thus, highly compact sensing configurations can be envisioned by using free-standing tape waveguides, and the feasibility to use sharp tape bends should greatly facilitate the access to narrow orifices or trenches for sensing purposes.

**Figure 7:**
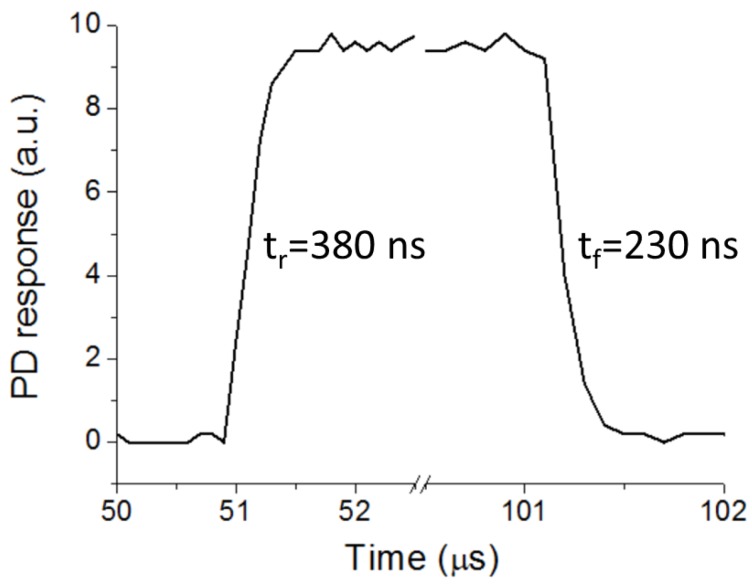
Time response of the PD in the optical interconnection of Figure 3 for 10 kHz TTL modulated LED. The 10%−90% rise time (t_r_) and fall time (t_f_) are 380 ns and 230 ns, respectively.

The principle of operation and/or the performance of some optical sensing schemes relies on the use of modulated optical signals [13]. It is therefore pertinent to study the dynamic response of the implemented optical waveguiding scheme. Figure 7 shows the PD time response of the 24.7-mm-long optical link (Figure 3) for a 10 kHz TTL modulated LED. The detected pulse exhibits 10%−90% rise and fall times of t_r_ = 380 ns and t_f_ = 230 ns, respectively. This corresponds to a bandwidth BW = 0.35/t_r_ = 921 kHz. These values coincide with those of the PD-LED system when the transmission medium is air (i.e., without tape waveguide interconnection), indicating that the time response of the tape-based configuration is limited by the optoelectronic components and associated circuitry, not by the tape waveguide. For sensing applications such as those based on phase-modulation fluorometry [14] the measured bandwidth is more than sufficient to allow proper operation.

### Liquid Detection Sensor

**Figure 8:**
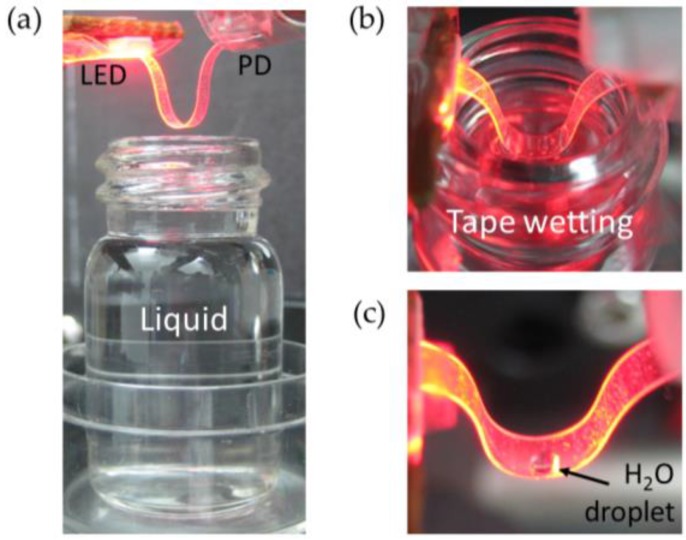
(**a**) Flexible tape-based optical interconnection configured for the refractive index sensing of liquids. When the bent waveguide is slightly immersed in a liquid contained in a small bottle (**b**), the optical confinement in the waveguide and, therefore, the detected power by the PD varies as a function of the liquid refractive index. The optical sensing configuration can detect even a tiny water droplet adhered to the convex side of the bent waveguide as that shown in (**c**).

The studied optical connection can be readily configured as an intrinsic intensity-based sensor for detecting the presence of liquids and measuring their refractive indexes. The principle of operation is based on the dependence of the tape waveguide bending losses with the refractive index (RI) of the surrounding medium. Figure 8a shows a photograph of the sensing configuration: the GC and tip end of a 24.7-mm-long tape waveguide are attached to the LED and PD, respectively, and the distance between them has been decreased in order to create a 180° bent tape waveguide.

To characterize the sensor performance, the bent waveguide was slightly immersed in different liquids (Figure 8b): methanol (RI = 1.326), ethanol (RI = 1.360), isopropanol (RI = 1.382) and cyclohexane (RI = 1.424), contained in small bottles. The closer the RI of the liquid to that of the tape (RI = 1.45, measured with reflectance interferometry), the larger the optical leakage into the surrounding medium, that is, the smaller the optical power detected by the PD. To assure the same interaction length, the immersion depth of the curved tape region into the different liquids was the same. This was achieved by keeping the tape configuration fixed and raising the open bottle underneath (Figure 8a), which contains the same liquid volume for all tested liquids, to a particular height through a z-axis translation stage. After testing a particular liquid, the tape was extracted from it by taking the bottle down, and then the power at the photodetector was monitored until it reached the air case value. The latter indicates that no or negligible liquid residues remain adhered on the tape surface because of their evaporation. Thus, the tape was ready to test another liquid.

**Figure 9:**
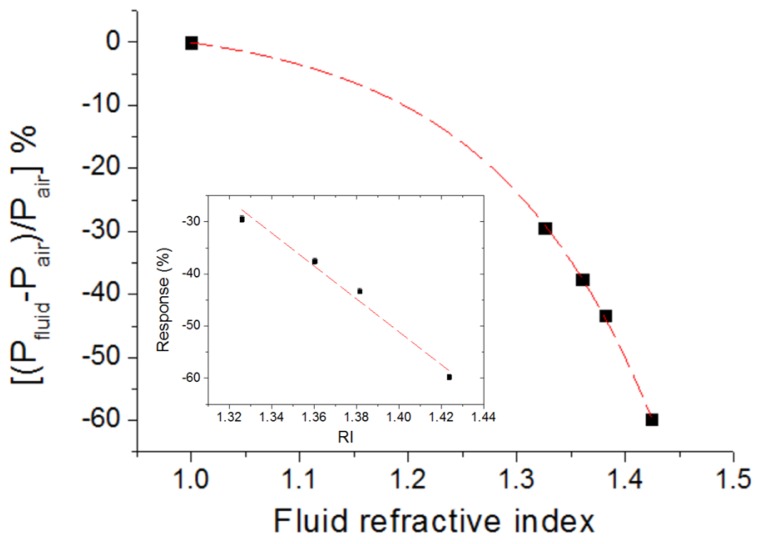
Relative variation of the detected power for the bent waveguide-based sensing configuration of Figure 8a as a function of the refractive index (RI) of the liquid that wets the bend. P_fluid_ and P_air_ are the detected powers when the bend is immersed in a fluid and air, respectively. The experimental data can be well fitted by an exponential function (dashed line) with adjusted R^2^ = 0.999. Error bars are equal or smaller than the dot size. Inset shows the measured values in the RI range 1.326–1.424 and a linear fit of the data (dashed line).

The measured calibration curve is plotted in Figure 9, which shows the relative variation of the detected power as a function of the refractive index of the liquids. The experimental data can be fitted by an exponential curve (adj. R^2^ = 0.999). Therefore, the derivative of the curve, i.e., the sensitivity, depends on the liquid RI and its absolute value increases as the RI increases. In particular, the sensitivity for RI values around 1.424 (cyclohexane) is −419 (%)/RIU, where RIU stands for refractive index unit. This means a RI resolution (limit of detection) of Δn = [0.3%/419 (%)/RIU] = 7 × 10^−4^ RIU, where the value 0.3% is the system response resolution, determined by the ratio of the minimum detectable power variation at the photodetector (measurement uncertainty): 0.1 µW, and the measured power for the air case P_air_ = 37.1 µW. Note that, according to Equation (1), the obtained minimum measurable power at the receiver end (P_PD_ = 0.1 µW) allows the use of tape lengths as long as 110 mm. If high accuracy is not critical or essential, as is the case for many engineering applications, the sensor response depicted in Figure 9 can be approximated by a linear function of slope −316 (%)/RIU (inset of Figure 9), which simplifies response data processing and interpretation.

Remarkably, the created optical sensing configuration was able to detect a small droplet of water adhered to the concave side of the bent waveguide as that shown in Figure 8c: a ~0.3 µL water droplet produced a relative detected power variation as large as −10.7%. This demonstrates the feasibility of the Scotch tape-based optical system for the ready detection of liquids.

## 4. Discussion

For the sake of comparison with other existing liquid refractive index sensing schemes, it should be mentioned that Abbe refractometers have a typical RI resolution in the range 1 × 10^−4^–2 × 10^−5^ RIU, however they are relatively bulky and expensive. Long-period fiber grating (LPFG) sensors based on resonant wavelength shifts exhibit a RI resolution of 1 × 10^−3^–2 × 10^−4^ RIU [15,16] but require bulky, expensive, and high precision instruments such as tunable laser sources and optical spectrum analyzers. Multi-D-shaped fiber sensors have demonstrated RI resolutions in the range 1 × 10^−3^–3 × 10^−4^ RIU by microstructuring the fiber using a femtosecond laser pulse technique [17]. An intrinsic intensity-based POF liquid detector sensor [18] was able to distinguish between water and oil (RI = 1.466), which, from Figure 9, can be clearly performed by the presented Scotch tape-based optical sensor. Thus the performance of the configuration studied here compares well to state-of-the-art intensity-based optical sensing devices despite its simplicity and low cost. The obtained RI resolution can be applicable to, for example, determining the sugar content in fruits, vegetables, juices, beverages, and in wine and beer making. In addition, cost-effectiveness favors disposability; therefore the presented sensing tape waveguide is particularly well-suited for single-use applications.

It should be noted that the tape waveguide structure has not been optimized for the highest sensitivity and significant improvements in this regard are expected by, for example, reducing the waveguide tape width, creating grooves [17] and/or perforating holes [19] in the sensing region in order to increase the interaction of the optical probe (guided light) with the environment, or by depositing metal films onto the tape surface to excite surface plasmons with the same purpose [20]. Note, however, that such sensitivity improvements are typically achieved at the expense of larger optical loss and fabrication complexity. Thus, trade-offs should be adopted when designing this type of sensors.

Besides increasing the sensitivity, the RI resolution of the sensor could be reduced by decreasing the system response resolution. The employed measurement equipment (digital multimeter) had a better resolution than the overall system response resolution; therefore, the latter must be mainly determined by the opto-electronic (LED and PD) and electronic (resistors) components and the DC power supply stability. Hence appropriate selection of low-noise optoelectronic and electronic components, together with a properly designed and implemented driver circuitry, should help to reduce the system measurement resolution and, consequently, the limit of detection of the studied refractometric sensor.

## 5. Conclusions

A cost-effective and easy-to-implement optical link between surface-normal optoelectronic devices based on a PSA tape waveguide for sensing applications has been proposed and demonstrated. The flexible optical connection is formed by directly attaching a waveguide end containing a metal GC onto a vertically emitting LED and the other waveguide termination (tip end) onto a photodetector. Large alignment tolerances, on the order of hundreds of microns, between the waveguide terminations and the corresponding optoelectronic devices have been obtained, which highly facilitate manual formation of optical connections. The overall coupling efficiency can be enhanced by 100% by just placing reflecting elements over the GC and tip terminals. The tape waveguide exhibits low bending loss and bend radius, which makes it well suited for creating sharp curves for compactness and/or easy accessibility purposes. The optical system exhibits a modulation BW of almost 1 MHz, which largely exceeds typical frequencies required for the operation of relevant sensing schemes based on optical modulation. The first sensing demonstration of the presented optical architecture has been shown through the easy implementation of an intrinsic, intensity-based, optical sensor for detecting liquids and measuring their refractive indexes, whose performance is well compared to intensity-based optical fiber sensors. The proposed configuration offers the possibility of creating low-cost, compact and easy-to-replace optical links by just sticking the waveguide terminations on the corresponding optoelectronic devices, which is ideal for sensing field applications requiring rapid on-site results.
